# Comparative psychological well-being evaluation among CKD patients on conservative therapy, dialysis, or kidney transplantation: a cross-sectional study

**DOI:** 10.3389/fpsyg.2025.1734424

**Published:** 2026-01-06

**Authors:** Patrizia Pignataro, Simona Simone, Manuela Dicarlo, Federica Cassone, Gianvito Caggiano, Raffaella Guido, Fausta Piancone, Carmen Sivo, Anna Maria Dipalma, Roberto Russo, Marco Spilotros, Pasquale Di Tonno, Silvia Colucci, Graziana Colaianni, Paola Pontrelli, Maria Grano, Loreto Gesualdo

**Affiliations:** 1 Department of Translational Biomedicine and Neuroscience (DiBraiN), University of Bari “A. Moro”, Bari, Italy; 2 Department of Precision and Regenerative Medicine and Ionian Area (DiMePRe-J)-Nephrology, Dialysis and Transplantation Unit, University of Bari "Aldo Moro", Bari, Italy; 3 Department of Precision and Regenerative Medicine and Ionian Area (DiMePRe-J), University of Bari “A. Moro”, Bari, Italy; 4 Department of Precision and Regenerative Medicine and Ionian Area (DiMePRe-J)-Urology, Andrology and Kidney Transplantation Unit, University of Bari "Aldo Moro", Bari, Italy

**Keywords:** psychological assessment, chronic kidney disease, kidney transplant, dialysis, irisin

## Abstract

Background Chronic kidney disease (CKD) has a significant impact on psychological well-being. Here, the psychological evaluation of patients with CKD undergoing replacement treatment (dialysis or kidney transplantation) and conservative therapy (preemptive patients, who are waiting list for kidney transplantation) was analyzed. In addition, serum irisin levels, a protein displaying anxiolytic and antidepressant effects in mice, were measured in dialysis patients. Methods Dialysis (*N* = 57), non-dialysis (preemptive, *N* = 31) and kidney transplant patients (*N* = 33) were enrolled. All participants underwent psychometric tests including State–Trait Anxiety Inventory (STAI-Y 1 and 2 form), Psychological General Well-Being Index (PGWBI), Symptom Checklist-90-R (SCL-90-R), etc. Serum irisin levels in dialyzed patients were measured by ELISA assay. Results Dialysis patient group scored worse on all tests performed than both preemptive and kidney transplant patients. Indeed, dialysis patients displayed the lowest PGWBI score, and higher scores of BDI, and STAY-1 and STAY-2, compared with preemptive and kidney transplant patients. We also found that about 40% of dialysis patients showed significant psychological distress with higher clinical attention values in the somatization, obsessive-compulsive, depression, and anxiety domains assessed by SCL-90. Furthermore, the stratification of all patients into groups younger and older than 50 years showed that the older group of transplanted patients displayed better outcomes than the younger ones. Finally, stratification of dialysis patients according to irisin levels revealed that only those with higher serum irisin levels had better psychological conditions in tests. Conclusions Kidney transplantation as well as conservative therapy were related to a lower prevalence of depressive symptoms and other psychological disorders than dialysis. Furthermore, all transplanted patients over 50 years of age showed better outcomes than the younger ones. However, dialyzed patients with high levels of circulating irisin displayed better psychological conditions. Overall, our findings supported the importance to provide timely access to transplantation and to improve psychological support for dialysis patients.

## 1 Introduction

Chronic kidney disease (CKD) is a widespread and progressively worsening condition affecting over 10% of the global population. CKD is defined by structural abnormalities of the kidneys or reduced renal function, as indicated by a Estimate Glomerular Filtration Rate (eGFR) of less than 60 mL/min/1.73 m^2^, present for more than 3 months, regardless of the underlying specific cause, with implications for overall health (Levey, 2022; Kovesdy, 2022; Webster et al., 2017). The main causes of CKD are diabetes and hypertension followed by glomerulonephritis and genetic diseases (Webster et al., 2017). Usually, the diagnosis is made when the symptoms worsen or after unexpected results from screening tests (blood or urine test strips) (Webster et al., 2017). Indeed, several individuals are often asymptomatic or have nonspecific symptoms. Sometimes people lose up to 90% of their kidney function before getting any symptoms (such as lethargy, itching, or loss of appetite), however they progress to a state defined as End-Stage Kidney Disease (ESKD) (Webster et al., 2017; Andrassy, 2013). CKD is classified into five stages based on the eGFR levels, which reflects the kidney damage severity (Levey et al., 2005). As a consequence of the disease reaching the fifth stage, people need artificial filtering called peritoneal dialysis and hemodialysis, or a kidney transplant (Lee et al., 2011). Currently, kidney transplantation represents the optimal treatment for end-stage kidney disease with significantly greater survival benefits and improved quality of life than CKD patients who remain on dialysis or are on a waiting list (Gonzalez-De-Jesus et al., 2011).

Considering the progressive course of CKD, the therapeutic and economic burden, and the prognostic implications, the health-related quality of life of CKD patients is significantly lower compared to the general population, and it decreases as eGFR declines (Webster et al., 2017). Indeed, depression, often associated with anxiety, is prevalent among individuals with CKD patients particularly those undergoing dialysis, with a significant impact on quality of life as well as morbidity and mortality (Simoes et al., 2019). Patients on the kidney transplant waiting list often experience a period of difficulty with symptoms of psychological distress and a significant increase in anxiety and depression with a progressive worsening during the wait (Corruble et al., 2010). Recently, it is highlighted the role of depression as an important risk factor for mortality and for transplant rejection for several reasons that may lead to lower adherence to the post-transplant treatment regimen, altered eating behavior, and the acquisition of sedentary lifestyles with inadequate levels of exercise (Dew et al., 2015).

Despite the apparent positive psychological impact of kidney transplantation in CKD patients, few comparative studies based on psychological assessment have been performed between patients treated with dialysis, non-dialysis patients, and those undergoing kidney transplantation.

Here, a psychological assessment was conducted on different groups of CKD patients undergoing conservative or replacement therapy. The term “conservative therapy” describes CKD preemptive patients awaiting kidney transplantation who are not on dialysis, receiving only standard nephrological care (such as antihypertensive and anemia therapy, metabolic control, lifestyle recommendations, personalized diet, *etc*.). Whereas “replacement therapy” refers both to dialysis (hemodialysis or peritoneal dialysis) and kidney transplantation. These replacement approaches are often complemented by specific medications and treatments, including anticoagulation and vascular access management in hemodialysis, peritonitis prophylaxis in peritoneal dialysis, and immunosuppressive therapy with infection prophylaxis in kidney transplant recipients (NICE, 2018; Martino et al., 2023).

In addition, only in dialysis patients, circulating irisin and its possible impact on psychological status was evaluated. This last investigation is supported by knowing that irisin, a circulating molecule produced by muscles during exercise, displays multiple beneficial effects on several target organs (Pignataro et al., 2021). Interestingly, it also exerts a neuroprotective role in cerebral areas involved in cognition and mood, and in animal studies showed anxiolytic and antidepressant effects (Pignataro et al., 2023; Pignataro et al., 2022; Dicarlo et al., 2023). Moreover, in mouse models systemic administration of irisin, which is able to cross the blood–brain barrier (Islam et al., 2021; Wrann et al., 2013), significantly increased the expression of the peroxisome proliferator-activated receptor-gamma coactivator (PGC-1alpha) and fibronectin type III domain-containing protein 5 (FNDC5), the precursor of irisin, in both the hippocampus and prefrontal cortex, two brain areas involved in depression (Dicarlo et al., 2023). Similarly, Siteneski et al. (2018) demonstrated that irisin administered into the cerebral lateral ventricles reduced depressive-like behaviors in mice undergoing stressful situations by modulating the expression of the brain-derived neurotrophic factor (BDNF), whose low levels have been associated to depression in humans (Brunoni et al., 2008).

Human studies have shown the relationship between irisin and depression. It has been observed that irisin levels are lower in depressed patients than in non-depressed individuals (Cicek et al., 2023; Tu et al., 2018). In addition, irisin levels in cerebrospinal fluid correlated negatively with the severity of depression (Goncalves et al., 2023). A recent systematic review and meta-analysis revealed that irisin levels in depressed subjects are lower than in non-depressed subjects. Of note, a negative correlation was shown between circulating irisin levels and depressive scores, indicating that the lower the circulating irisin level, the more severe the depressive symptoms (Han et al., 2025).

Therefore, the present study aimed to monitor the psychological well-being of CKD patients by evaluating in parallel whether circulating levels of irisin, exerting anti-depressant activity, may impact their psychological status.

## 2 Methods

### 2.1 Participants

A total of 120 patients were enrolled in this cross-sectional study, 87 with diagnosis of CKD and 33 transplanted, divided as follows: non-dialysis patients (preemptive, who are on waiting list for kidney transplantation) with stage 4 or 5 CKD (*N* = 31), dialysis patients (*N* = 56) including 30 on hemodialysis and 26 on peritoneal dialysis, and kidney transplant recipients (*N* = 33). All patients were recruited at the Nephrology, Dialysis, and Transplantation Unit of University of Bari.

Patients were selected according to the following inclusion/exclusion criteria:

### 2.2 Inclusion criteria

- Patients in the fourth or fifth stage of CKD;
- CKD diagnosis confirmed at least 6 months prior to enrollment;
- patients undergoing hemodialysis/peritoneal dialysis;
- kidney transplant recipients;
- ages > 18 to < 80 years.

### 2.3 Exclusion criteria

- Diagnosis of Acute kidney injury (AKI);
- recent nephrotoxicity;
- hearing/visual/language impairment;
- diagnosis of psychiatric disorders;
- alcohol and/or drug abuse;
- patients on their second or third transplant.

The stage of CKD patients was determined using the Estimated Glomerular Filtration Rate (eGFR), calculated from serum creatinine levels via the Chronic Kidney Disease-Epidemiology Collaboration (CKD-EPI) formula. Staging was conducted by Nephrologists from the Nephrology, Dialysis and Transplantation Unit of the University of Bari following the Kidney Disease: Improving Global Outcomes (KDIGO) international guidelines (Levey et al., 2005; Stevens and Levin, 2013).

This study was authorized by the local ethical committee (Protocol 1845/CEL; verbale Prot. N.658, September 12, 2024), in accordance to the Declaration of Helsinki (World Medical Association, 1991). Each patient gave their informed written consent. There was no discrimination against participants based on their age, gender, or ethnicity; enrollment in this study was solely based on their clinical diagnosis.

Demographic data included age, sex, years, physical exercise, and Body Max Index (BMI) are summarized in Table 1.

**Table 1:** The demographic characteristics of patients.

Variable	Preemptive patients (*n* = 31)	Dialysis patients (*n* = 56)	Transplant patients (*n* = 33)	*p*-value
Sex, *n* (%)				ns
Male	19 (61%)	36 (64%)	18 (55%)	
Female	12 (39%)	20 (36%)	15 (45%)	
Age, mean (±SD)	51 (±15)	58 (±15)	54 (±14)	ns
Physical exercise, *n* (%)	10 (32%)	18 (32%)	11 (33%)	ns
BMI, mean (±SD)	23.3 (±3.3)	25.17 (±4.6)	24.8 (±3.9)	ns

### 2.4 Psychological assessment

Each patient underwent a psychological interview with administration of psychometric tests. To evaluate the psychological characterization of the population, a comprehensive assessment was performed using 6 psychometric tests. In particular, Psychological General Well-Being Index (PGWBI) rated overall subjective health-related quality of life (Grossi et al., 2006; Dupuy, 1984), Beck’s Depression Inventory (BDI) evaluated depressive symptoms (Beck et al., 1996; Ghisi et al., 2006), State–Trait Anxiety Inventory—Form Y 1 and 2 (STAI-I and STAI-II) measured anxiety levels (Spielberger, 1983; Pedrabissi and Santinello, 1989), and Toronto Alexithymia Scale (TAS-20) assessed awareness and regulation of emotions (Bagby et al., 1994; Bressi et al., 1996). Symptom Checklist-90-R (SCL-90-R) examined general psychological distress (Sarno et al., 2011; Derogatis, 1977; Prunas et al., 2012). Details of the psychometric tests are summarized in Table 2.

**Table 2:** Psychometric instrument characteristics administered to the study participants.

Scale	Author(s), year	Italian validation/year	Subscales	Number of items	Score range	Cronbach’s alpha (for Italian version)
State–Trait Anxiety Inventory-I (STAI-I)	Spielberger (1983)	Pedrabissi and Santinello (1989)	State anxiety	20	20–80	0.91 (total score)
State–Trait Anxiety Inventory-II (STAI-II)	Spielberger (1983)	Pedrabissi and Santinello (1989)	Trait anxiety	20	20–80	0.85–0.90 (total score)
Beck’s Depression Inventory (BDI)	Beck et al. 1996	Ghisi et al. (2006)	Sadness, pessimism, failure, loss of pleasure, guilt, feelings of punishment, low self-esteem, self-criticism, suicidal thoughts, crying, agitation, loss of interest, indecision, feelings of uselessness, loss of energy, sleep disturbances, irritability, appetite disturbances, concentration difficulties, fatigue, sex	21	0–63	0.90 (total score)
Symptom Checklist-90-R (SCL-90-R)	Derogatis (1977)	Prunas et al. (2012)	Somatization, obsessive-compulsive, interpersonal sensitivity, depression, anxiety, hostility, phobic anxiety, paranoid ideation, psychoticism Global indexes	90	0–4 per item	0.70–0.96 (per domain)
Psychological General Well-Being Index (PGWBI)	Dupuy (1984)	Grossi et al. (2006)	Anxiety, depressed mood, positive well-being, self-control, general health, vitality	22	0–110	0.90 (total score)
Toronto Alexithymia Scale (TAS-20)	Bagby et al. (1994)	Bressi et al. (1996)	Difficulties identifying feelings, difficulties describing feelings, externally oriented thinking	20	20–100	0.81–0.86 (total score)

### 2.5 Sample collection

For the collection of serum, all patients underwent venous blood sampling via venipuncture. Serum samples were collected in separator tubes. After clotting at room temperature for 30 min, samples were centrifuged at 1000 g (rcf) for 20 min. Serum samples were aliquoted in polypropylene tubes and frozen at −80° C until use.

### 2.6 Serum Irisin assay

Serum irisin levels were measured by the competitive enzyme-linked immunosorbent assay (ELISA) kit (EK-067-29, Phoenix Pharmaceuticals, Burlingame, CA, USA). The sensitivity of the kit was 1.29 ng/mL and the detection range was 0.1–1,000 ng/mL. Inter-assay and intra-assay variation of <15 and <10%, respectively. According to the manufacturer’s instruction standard dilutions, positive controls, and patient samples were analyzed in duplicate. Absorbance at 450 nm was measured by a plate reader (Eon, BioTek, Winooski, Vermont). Results were reported in nanograms per milliliter.

### 2.7 Statistical analysis

Statistical analysis was carried out using GraphPad Prism software (Version 9.5.0; GraphPad Software, San Diego, CA). Data were subjected to the Shapiro–Wilk normality test to evaluate the sample distribution. For normally distributed values, ANOVA with Tukey’s multiple comparison tests was performed, and for non-normally distributed values, Kruskal-Wallis multiple comparison test was used. Values were considered statistically significant at *p* < 0.05.

## 3 Results

### 3.1 Patient global psychological assessment

**Figure 1:**
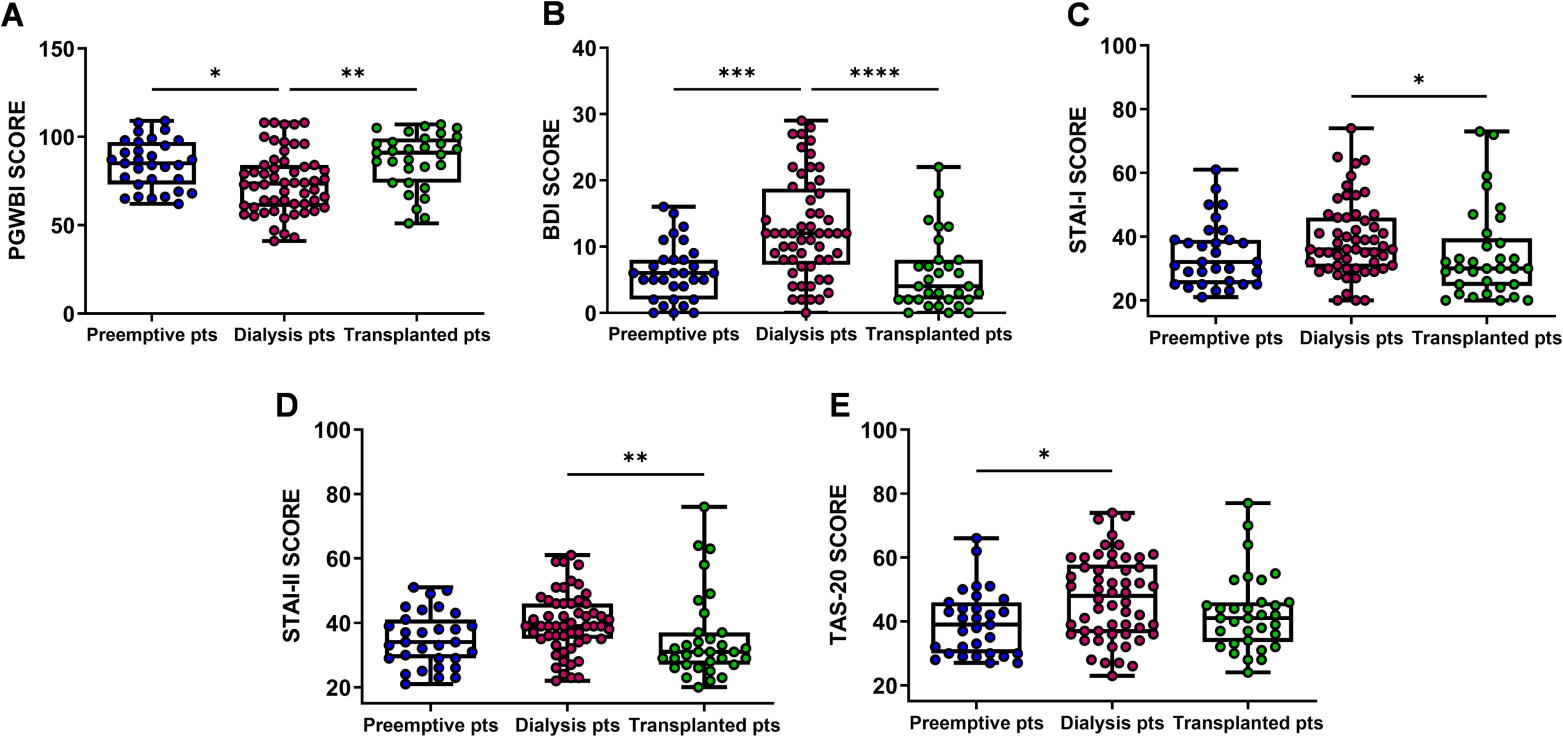
Comparison of Psychological General Well-Being Index (PGWBI) **(A)**, Back’s Depression Inventory (BDI) **(B)** State–Trait Anxiety Inventory—Form I and II (STAI-I and STAI-II) **(C,D)**, Toronto Alexithymia Scale (TAS-20) **(E)** scores among Preemptive, Dialysis and Transplanted patients. Data are presented as box-and-whisker plots with median and interquartile range, from maximum to minimum, with all data points shown (ANOVA/Tukey’s multiple comparison test or Kruskal–Wallis test/Dunn’s multiple comparison test). *p < 0.05; **p < 0.01; ***p < 0.001; ****p < 0.0001. pts., patients.

Dialysis patients showed a significant reduction in general psychological well-being (PGWBI score) and an increase in the severity of depressive symptoms (BDI score) compared with both preemptive (PGWBI *p* = 0.016; BDI *p* = 0.0003) and kidney transplant patients (PGWBI *p* = 0.002; BDI *p* = 0.0001) (Figures 1A,B). We also observed that dialysis patients displayed higher anxiety levels, measured as state and trait anxiety (STAI-I and STAI-II scores, respectively), compared with kidney transplant patients (STAI-I1 *p* = 0.04; STAI-II *p* = 0.005) (Figures 1C,D). Additionally, the same patients showed a significant increase in difficulty in identifying and describing emotions (TAS-20 score), compared with preemptive patients (TAS-20 *p* = 0.017) (Figure 1E).

### 3.2 Global psychological assessment in younger and older patients

**Figure 2:**
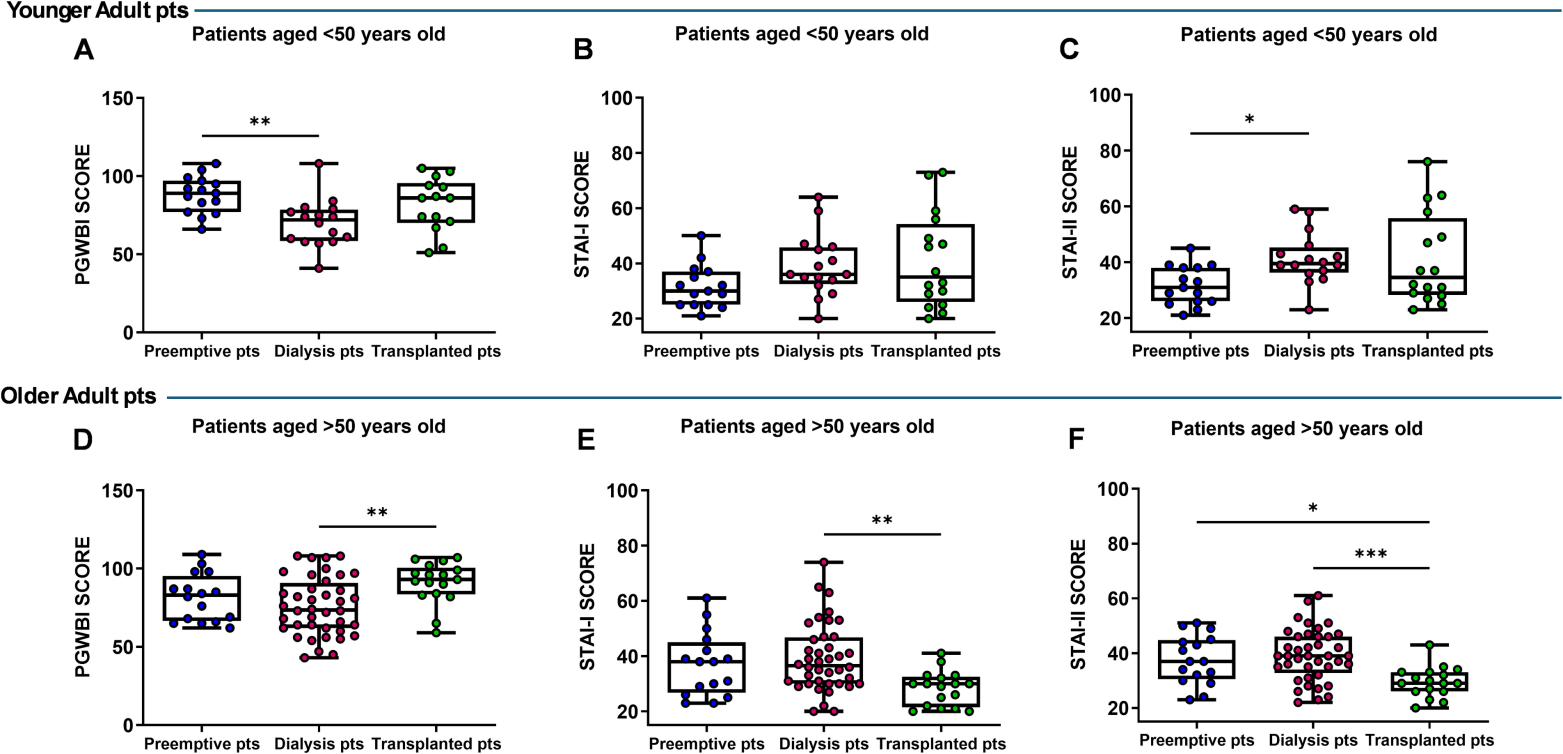
Comparison of Psychological General Well-Being Index (PGWBI) **(A,D)**, State–Trait Anxiety Inventory—Form I (STAI-I) **(B,C)** and II (STAI-II), **(E,F)** scores in preemptive, dialysis, and transplanted younger (<50 years) and older patients (>50 years). Data are presented as box-and-whisker plots with median and interquartile range, from maximum to minimum, with all data points shown (ANOVA/Tukey’s multiple comparison test or Kruskal–Wallis test/Dunn’s multiple comparison test). *p < 0.05; **p < 0.01; ***p < 0.001. pts., patients.

Patients were stratified into groups with age lower (younger adult patients) or higher (older adult or patients) than 50 years. It was found that the dialysis patients under 50 years of age showed a worse level of general well-being and higher trait anxiety as evidenced by the values of PGWBI (*p* = 0.004) and STAI-II (*p* = 0.021) that were significantly different *vs.* the preemptive group, and only for PGWBI reached a trend near to significance toward transplant recipients (Figures 2A,C). No marked differences in STAI-I were observed among the three groups of patients (Figure 2B). However, when we considered the group of older adult dialysis patients, we found that they showed significantly different and worsening values of PGWBI (*p* = 0.007), STAI-I and STAI-II (*p* = 0.002; *p* = 0.0006) compared with the transplant group. No differences emerged when these scores were compared with the preemptive patients (Figures 2D–F).

**Figure 3:**
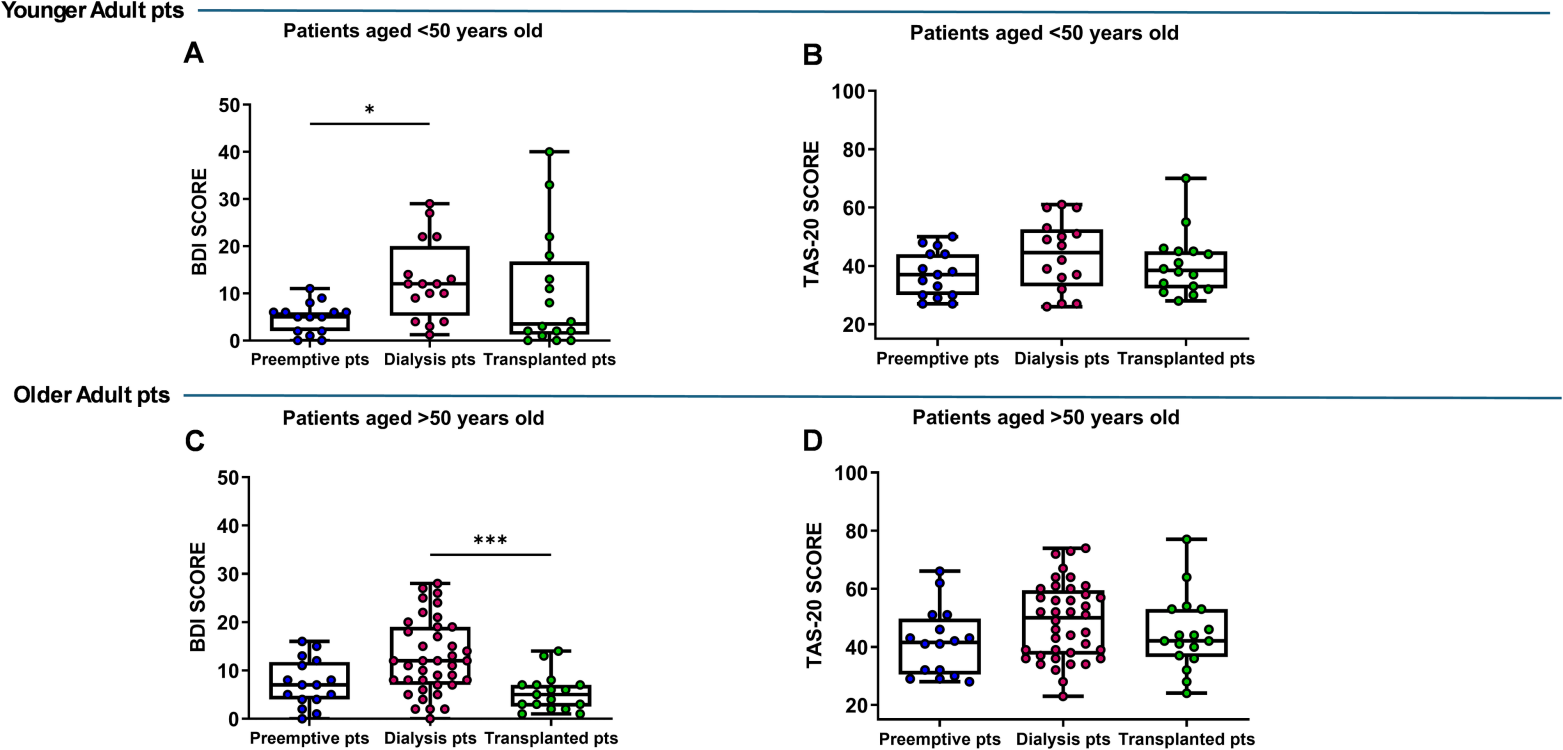
Back’s Depression Inventory (BDI) **(A,C)**, Toronto Alexithymia Scale (TAS-20) **(B,D)** scores in preemptive, dialysis and transplanted younger (<50 years) and older patients (>50 years). Data are presented as box-and-whisker plots with median and interquartile range, from maximum to minimum, with all data points shown (ANOVA/Tukey’s multiple comparison test or Kruskal–Wallis test/Dunn’s multiple comparison test). *p < 0.05; ***p < 0.001. pts., patients.

In addition, the BDI in younger dialysis patients was significantly higher than in the preemptive group (*p* = 0.026), while older dialysis patients showed significantly higher BDI score than transplant patients (*p* = 0.0008) (Figures 3A,C). No difference in TAS-20 score was observed in the analyzed groups (Figures 3B,D).

### 3.3 Psychological assessment of dialysis patients stratified by low and high irisin serum levels

Serum irisin levels were measured in the dialysis patient group and we found that they were distributed over a wide range (data not shown). Therefore, dialysis patients were then divided into two groups, low (under the median value) and high irisin (over the median value). Based on this stratification, we compared the psychological parameters of low- and high-irisin dialysis patients with those of preemptive and transplanted patients.

**Figure 4:**
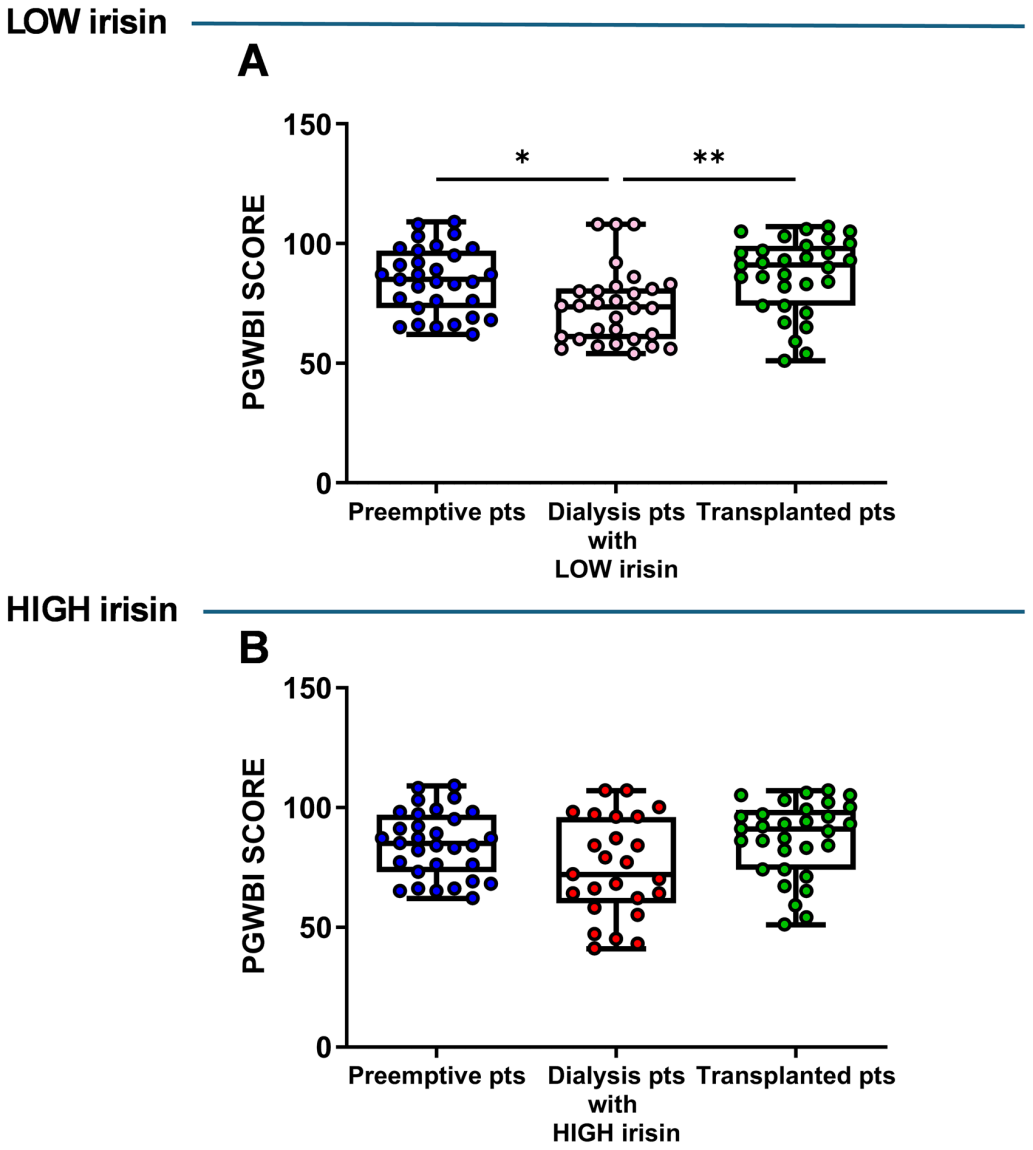
Comparison of psychological General Well-Being Index (PGWBI) score in low **(A)** and high **(B)** irisin dialysis patients versus total preemptive and transplanted patients. Data are presented as box-and-whisker plots with median and interquartile range, from maximum to minimum, with all data points shown (ANOVA/Tukey’s multiple comparison test or Kruskal–Wallis test/Dunn’s multiple comparison test). *p < 0.05; **p < 0.01. pts., patients.

The results showed that, in dialysis patients with low irisin levels, the overall psychological well-being (PGWBI score) was significantly lower than in preemptive and transplant patients (preemptive vs. dialysis, *p* = 0.014; transplanted vs. dialysis, *p* = 0.002) (Figure 4A). Conversely, no significant differences were observed in dialysis patients with high irisin levels compared with both preemptive and transplanted patients (Figure 4B).

**Figure 5:**
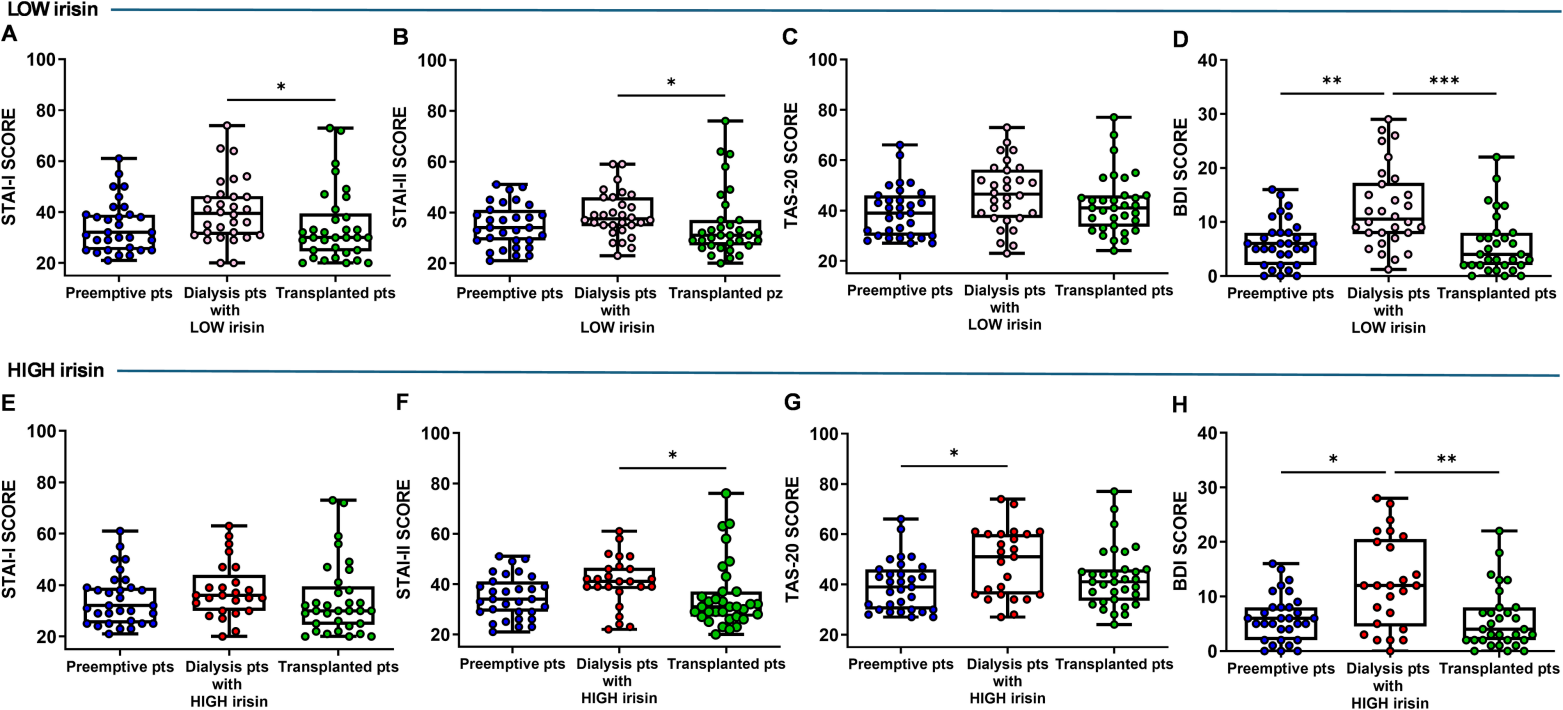
Comparison of State–Trait Anxiety Inventory—Form I and II (STAI-I and STAI-II) **(A,B,E,F)**, Toronto Alexithymia Scale (TAS-20) **(C,G)**, Back’s Depression Inventory (BDI) **(D,H)** scores in low (upper panel) and high (bottom panel) irisin dialysis patients versus total preemptive and transplanted patients. Data are presented as box-and-whisker plots with median and interquartile range, from maximum to minimum, with all data points shown (ANOVA/Tukey’s multiple comparison test or Kruskal–Wallis test/Dunn’s multiple comparison test). *p < 0.05; **p < 0.01; ***p < 0.001. pts., patients.

Moreover, in the subgroup with low irisin levels, dialysis patients showed higher STAI-I (*p* = 0.037) and STAI-II scores (*p* = 0.025) compared with transplanted patients (Figures 5A,B), no difference in TAS-20 score between patient groups (Figure 5C) and high BDI score compared with both preemptive and transplanted patients (*p* = 0.0014; *p* = 0.0001) (Figure 5D). In the subgroup with high irisin levels, dialysis patients showed no difference in STAI-I score compared with the two other groups (Figure 5E) and a greater STAI-II score (*p* = 0.016) compared with transplanted patients (Figures 5E,F). Furthermore, these patients also showed higher TAS-20 score (*p* = 0.040) than preemptive ones (Figure 5G) and higher BDI score than both preemptive (*p* = 0.023) and transplanted patients *p* = 0.0033 (Figure 5H).

### 3.4 General psychological distress

Concerning the results of Symptoms Checklist-90-Revised (SCL-90-R), Table 3 shows the percentage of patients with an altered SCL-90 score (i.e., with individual scores > 1), which specifically included 8 of 31 preemptive patients, 23 of 56 dialysis patients, and 9 of 33 transplanted patients.

**Table 3:** Percentage of patients with altered SCL-90-R domains (%).

SCL-90-R domains	Groups		
	Preemptive patients *N* = 8/31	Dialysis patients *N* = 23/56	Transplanted patients *N* = 9/33
Somatization (SOM)	87.5%	73.91%	55.55%
Obsessive-Compulsive (O-C)	37.5%	56.52%	77.77%
Interpersonal sensitivity (I-S)	0%	21.73%	66.66%
Depression (DEP)	25.0%	56.52%	44.44%
Anxiety (ANX)	12.5%	39.13%	55.55%
Hostility (HOS)	0%	26.08%	44.44%
Phobic anxiety (PHOB)	12.5%	13.04%	44.44%
Paranoid ideation (PAR)	12.5%	30.43%	55.55%
Psychoticism (PSY)	0%	21.73%	44.44%
Global Severity Index (GSI)	12.5% (1 out of 8)	39.13% (9 out of 23)	77.77% (7 out of 9)

SCL-90-R, Symptom Checklist-90-Revised.

Regarding the overall psychological distress (measured by Global Score Index, GSI), we found that GSI score was low in preemptive patients (12.5%; only one of 8 with GSI > 1), it increased in dialyzed (39.13%; 9 patients of 23 with a GSI > 1), and became severe in the transplant recipients (77.77%; 7 of 9 patients with a GSI > 1) aged less than 50 years.

Dialysis patients with significant psychological distress showed higher clinical attention values in the SCL-90 test in the following domains: somatization (73.91%), obsessive-compulsive (56.52%), depression (56.52%), and anxiety (39.13%). Paranoia (30.43%), hostility (26.08%), interpersonal sensitivity (21.73%), psychoticism (21.73%), and phobia-related anxiety (13.04%) were fewer common disorders.

Transplanted patients with a significant psychological distress showed higher clinical attention values in the SCL-90 test in the following domains: obsessive-compulsive (77.77%), interpersonal sensitivity (66.66%), anxiety (55.55%), somatization (55.55%), and paranoia (55.55%) while in a reduced percentage depression (44.44%), hostility (44.44%), psychoticism (21.73%), and phobia anxiety (13.04%).

## 4 Discussion

Chronic kidney disease is a condition that affects more than 10% of the world’s population and improving their quality of life is one of the priorities for their clinical management. This study examined the psychological assessment of patients with chronic kidney disease undergoing conservative or replacement therapy distinguished in preemptive, dialyzed, and kidney transplant patients. Moreover, we also studied a possible influence of irisin, a multifunctional protein exerting beneficial effects on body homeostasis (Boström et al., 2012; Sarno et al., 2011; Bagby et al., 1994; Bagby et al., 1994) displaying anxiolytic and antidepressant effects in mice (Pignataro et al., 2023; Pignataro et al., 2022), with the psychological status of dialysis patients who showed the worst psychological condition. Here we show that the dialysis patient group scored worse in all tests performed than the other two groups indicating that CKD patients undergoing dialysis are those with a lower level of general well-being and more prone to developing depressive symptoms and anxiety.

The symptoms of depression and anxiety, measured by BDI and STAI-I and STAI-II, were significantly higher in dialysis patients than non-dialysis (preemptive) patients, awaiting transplantation, and transplant recipients. These findings are consistent with prior works reported in the literature, although different psychometric tests were used (Dziubek et al., 2021; Zaragoza-Fernandez et al., 2025).

Further results from the assessment of general distress (assessed by the SCL-90), revealed that about 40 percent of dialysis patients showed significant psychological distress with higher clinical attention values in the domains of somatization, obsessive-compulsive, depression and anxiety. These data are consistent with a recent study in which the SCL-90 questionnaire was used to assess patients undergoing hemodialysis and peritoneal dialysis. In this study, the authors clearly demonstrated the high level of distress experienced by these patients and highlighted their increased risk of developing somatization, depression, and anxiety disorders (Yilmaz et al., 2021).

As expected, when we analyzed PGWBI used to measure general well-being status, it emerged that dialysis patients have the lowest levels of PGWBI compared to both preemptive and transplanted patients.

On the other hand, dialysis is time-consuming treatment, and as the condition worsened, patients may experience overall distress not only from kidney disease complications but also from the time spent on dialysis therapy that affects their daily activities, and the frustration of feeling like a burden to their family (Bakewell et al., 2002). A two-year study on peritoneal dialysis patients revealed a significant decrease in quality of life related to health, with more significantly altered domains in general health issues, emotional well-being, renal disease risk, and patient satisfaction (Bakewell et al., 2002).

Interestingly, we found differences in psychological condition related to the age of patients highlighting that transplanted patients aged more than 50, showed better outcomes than the younger ones. The better outcomes of the older group may be explained by the fact that it includes individuals who have retired or otherwise had their working lives already settled down. Moreover, the dialysis patients aged <50 years had the worst psychological conditions (poorer health-related psychological well-being and higher trait anxiety levels) compared to preemptive patients. This could be due to the fact that younger dialysis patients are in the middle of the working life and are being forced to change several aspects of their lives.

These results agree with a recent study comparing dialysis patients and preemptive patients over 65 years old on the waiting list, in which it has been reported that dialysis patients have significantly lower scores than preemptive patients in the Physical Functioning and Role Physical dimensions (generic domains of the Kidney Disease Quality of Life Short Form, KDQOL-SF, questionnaire) (Lonning et al., 2018).

Considering that dialysis patients, especially younger ones, are in a worse psychological condition we measured the levels of circulating irisin, a multifunctional protein secreted by skeletal muscle during exercise, involved in several disorders including CKD (Li and Lindholm, 2024) and exerting beneficial effects on body homeostasis (Boström et al., 2012). By stratifying these patients according to the levels of this protein, we found that dialyzed patients with higher serum levels of irisin were those with better psychological conditions, such that their scores on various psychological tests were no longer significantly different from those in the preemptive and transplanted groups.

Irisin has been recently involved in neural plasticity, and the studies in animal models exposed to stress indicate that irisin is involved in modulating depressive-like behaviors by regulating the expression of neurotrophic factors in the hippocampus and prefrontal cortex of the brain (Pignataro et al., 2023; Pignataro et al., 2022; Dicarlo et al., 2023). As a result, this myokine might represent a molecular link between kidney disease and the patient’s psychological status that might warrant further investigation.

In conclusion, these findings indicate that kidney transplantation as well as conservative therapy are related to a lower prevalence of depression and other psychological disorders than dialysis. Moreover, the results support the importance of providing timely access to transplantation and improving psychological support for dialysis patients.

### 4.1 Limitations

It is important to acknowledge the limitations of this study. Firstly, its single-center design. Secondly, its cross-sectional nature with a lack of longitudinal follow-up. Third, levels of irisin were exclusively analyzed in dialysis patients considering their psychological status; it would be interesting to evaluate the myokine levels in all groups of individuals considered. Fourth, more research is required to elucidate the relationship between irisin concentration and psychological assessment. Finally, there is the presence of potential residual confounding factors that could not be fully measured or controlled. These include, for example, differences in inflammatory status, treatment adherence variability, differences in dialysis protocols, nutritional factors, levels or types of physical activity, and socioeconomic or psychosocial variables.

In addition, it would be desirable in the future to assess the global cognitive status in order to evaluate the efficiency of single domains (memory, attention, executive functions, language, and visuospatial abilities) of this population considering the link between cognitive health and chronic kidney disease.

## Data Availability

The raw data supporting the conclusions of this article will be made available by the authors, without undue reservation.
